# CDK9 binds and activates SGK3 to promote cardiac repair after injury via the GSK-3β/β-catenin pathway

**DOI:** 10.3389/fcvm.2022.970745

**Published:** 2022-08-23

**Authors:** Jiateng Sun, Tongtong Yang, Tianwen Wei, Liuhua Zhou, Tiankai Shan, Jiawen Chen, Lingfeng Gu, Bingrui Chen, Liu Liu, Qiqi Jiang, Chong Du, Yao Ma, Hao Wang, Feng Chen, Xuejiang Guo, Yong Ji, Liansheng Wang

**Affiliations:** ^1^Department of Cardiology, The First Affiliated Hospital of Nanjing Medical University, Nanjing, China; ^2^Department of Biostatistics, School of Public Health, China International Cooperation Center for Environment and Human Health, Nanjing Medical University, Nanjing, China; ^3^State Key Laboratory of Reproductive Medicine, Department of Histology and Embryology, Nanjing Medical University, Nanjing, China; ^4^Key Laboratory of Cardiovascular and Cerebrovascular Medicine, Collaborative Innovation Center for Cardiovascular Disease Translation, Nanjing Medical University, Nanjing, China; ^5^Key Laboratory of Targeted Intervention of Cardiovascular Disease, Collaborative Innovation Center for Cardiovascular Disease Translation, Nanjing Medical University, Nanjing, China

**Keywords:** myocardial regeneration, myocardial infarction, CDK9, cardiac repair, GSK-3β/β-catenin pathway

## Abstract

The mammalian heart possesses entire regeneration capacity after birth, which is lost in adulthood. The role of the kinase network in myocardial regeneration remains largely elusive. SGK3 (threonine-protein kinase 3) is a functional kinase we identified previously with the capacity to promote cardiomyocyte proliferation and cardiac repair after myocardial infarction. However, the upstream signals regulating SGK3 are still unknown. Based on the quantitative phosphoproteomics data and pulldown assay, we identified cyclin-dependent kinase 9 (CDK9) as a novel therapeutic target in regeneration therapy. The direct combination between CDK9 and SGK3 was further confirmed by co-immunoprecipitation (Co-IP). CDK9 is highly expressed in the newborn period and rarely detected in the adult myocardium. *In vitro*, the proliferation ratio of primary cardiomyocytes was significantly elevated by CDK9 overexpression while inhibited by CDK9 knockdown. *In vivo*, inhibition of CDK9 shortened the time window of cardiac regeneration after apical resection (AR) in neonatal mice, while overexpression of CDK9 significantly promoted mature cardiomyocytes (CMs) to re-enter the cell cycle and cardiac repair after myocardial infarction (MI) in adult mice. Mechanistically, CDK9 promoted cardiac repair by directly activating SGK3 and downstream GSK-3β/β-catenin pathway. Consequently, our study indicated that CDK9 might be a novel target for MI therapy by stimulating myocardial regeneration.

## Introduction

Myocardial infarction (MI) and subsequent heart failure are the leading causes of morbidity and mortality in patients with cardiovascular disease (CVD) worldwide (1). Therefore, myocardial regenerative therapy for MI has become a research hotspot in recent years, and the discovery of the neonatal myocardial regeneration period provides a new theoretical basis for studying endogenous myocardial regeneration (2). Neonatal mice can achieve complete functional and structural repair by endogenous myocardial regeneration within 7 days after MI or apical resection (AR), but this ability is rapidly lost (3). Therefore, the relevant studies based on the regulation of the myocardial regeneration period can indicate the return of mature cardiomyocytes (CMs) to the cell cycle after injury to achieve myocardial regeneration and repair.

The cyclin-dependent kinases (CDKs) family has a remarkable role in promoting proliferation. CDKs control critical cell cycle checkpoints and transcriptional events in response to extracellular and intracellular signals leading to mammalian cell proliferation. The intervention of CDKs has been a hot topic in cancer therapy, among which inhibitors that block the activity of CDK4 and CDK6 enzymes (CDK4/6) have been approved by the FDA to treat metastatic hormone receptor-positive breast cancer (4). Positive cell cycle regulators such as cyclin D1, CDK4/6, Rb, and E2F transcription factors are highly expressed in fetal CMs but significantly down-regulated in neonatal and adult CMs, consistent with postnatal CMs cycle arrest (5). DiStefano et al. found that neonatal and adult CMs could re-enter the cell cycle by knocking out endogenous CDKs inhibitors (6). The results suggest that neonatal and adult CMs can achieve proliferative capacity by regulating CDKs key nodes. Recently, the combined expression of CDK1, CDK4, cyclin B1, and cyclin D1 can induce nuclear replication and division in mice, rats, and human cardiac muscle and achieve cardiac proliferation through stable cytoplasmic separation (7).

The role of protein kinases in CVD has also gradually gained attention (8, 9). Cyclin-dependent kinase 9 (CDK9) is the most representative member of the CDKs family and plays an essential role in the development and progression of cardiac hypertrophy (10). Sano et al. found that the expression of CDK9 was upregulated, and its activity increased during myocardial hypertrophy, while chronic activation of CDK9 led to heart failure in the myocardium of adult mice (11). Besides, CDK9 could be a potential biomarker of atherosclerosis, which significantly increases in the serum of patients with CVD, and plays a vital role in inflammatory diseases (12). In addition, CDK9 inhibitor LDC000067 and siRNA-mediated CDK9 knockdown could reverse low-density lipoprotein-induced inflammation and phenotypic conversion from contractile to synthetic phenotypes of human vascular smooth muscle cells (HVSMCs) by inhibiting the NF-kB signaling pathway in HVSMCs (13). As a core component of positive transcription elongation factor b, CDK9 has been involved in differentiating mice embryonic stem cells into CMs by interacting with GATA4 (14).

Our previous study found that serine/threonine-protein kinase 3 (SGK3) remains highly expressed during the neonatal stage (myocardium with regenerative capacity), consistent with the mammalian myocardial regenerative period. Additionally, overexpression of SGK3 kinase induces proliferation of CMs by mediating the GSK-3β/β-catenin signaling pathway, protecting against myocardial I/R injury (9). In exploring the mechanism of SGK3 in myocardial protection, we surprisedly found that CDK9 and SGK3 directly bind, and CDK9 could act as the upstream regulator of SGK3 expression, which may play a more significant role in promoting CMs proliferation. Therefore, CDK9 warrants further investigation of the relevant effects as a therapeutic target on CMs proliferation in mammals.

## Materials and methods

### Mice

Neonatal 1-day-old and 8-week-old mice were used in our study. Institute of Cancer Research (ICR) mice were purchased from the Animal Core Facility of Nanjing Medical University and raised in the specific pathogen-free (SPF) environment with 12 h dark and 12 h light cycle. The mice were all healthy and had free access to water and food. As shown below, mice received AR at 1 day of age and MI at 8 weeks of age (All experiments involving animals are carried out following the Guidelines for the Use and Care of Laboratory Animals. All animal protocols have been approved by the Animal Care and Use Committee of Nanjing Medical University).

### Apical resection and apical intra-myocardial microinjection

AR surgery was performed on neonatal mice 1 day after birth. P1 mice were anesthetized on an ice bed for 3–4 min. The skin was cut from the left fourth intercostal space by microsurgery. The heart was squeezed out of the chest by pressing the abdomen of the mice. Surgical scissors were then used to remove 10–15% of the apex tissue from the left ventricle. After surgery, the heart was gently returned to the chest, and the thoracic wall and skin incision were closed immediately using 6-0 sutures. Finally, mice were placed on the thermostat to recover their body temperature quickly and put back into the cage after returning to normal activities. The sham-operated mice were subjected to the same procedure mentioned above without AR. All mice were sent to their mothers after surgery. After the apex of the heart is partially resected, Ad5: cTnT-CDK9i (The target sequence of shRNA for mice CDK9 was 5′-GGTCACCAAGTACGAGAAACT-3′) or Ad5: cTnT-CONi (the total amount of virus injected was 1*10^9^ PFU) was injected around the apex of the P1 heart by a microsyringe with a 36G needle as we previously described (15). Briefly, the virus dilution was pre-dyed by Trypan blue and injected to the ventricular myocardium of AR border zone. Blue staining of the marginal myocardium indicates a successful intra-myocardial injection.

### Myocardial infarction and apical intra-myocardial microinjection

Male mice aged 8 weeks (P56) were anesthetized by intraperitoneal injection of 1.2% Avertin (Sigma-Aldrich, St. Louis, MO, United States) and artificially ventilated by a small animal ventilator. Ophthalmic scissors were used to cut the skin along with the left fourth costal space. The intercostal muscles were bluntly separated by ophthalmic forceps and then entered the chest to expose the left atrial appendage. The LAD was ligated with a 6-0 suture needle from the lowest margin of the left atrial appendage. Then the intercostal muscle and skin incision were sutured layer by layer with 3-0 sutures. After the operation, the mice were placed on the thermostat to wake up. The sham-operated mice were subjected to the same procedure mentioned above without MI. After LAD was ligated, adenovirus AAV9: cTnT-CDK9 or AAV9: cTnT-CON was injected around the apex of P56 using a microsyringe with a 36G needle as previously described (15) (the total virus dose was 1.5*10^9^ v.g.). Briefly, the virus dilution was pre-dyed by Trypan blue and injected into the ventricular myocardium of the infarct border zone, defined as the myocardial tissue 1 ∼ 2 mm away from the infarction edge. Blue staining of the marginal myocardium indicates a successful intra-myocardial injection.

### Echocardiography

Cardiac function was determined by echocardiography Vevo 2100 (VisualSonics, Ontario, CA, United States) following AR/MI injury at the designed timepoint (1/22 day-post-AR, *n* = 7; 4/28 day-post-MI, *n* = 8). After aligning the transverse B-mode with the papillary muscles, cardiac function was measured on M-mode images. The left ventricular contractile function parameters (LVEF/LVFS) were automatically calculated using the accompanying software.

### Histological staining and assessment of infarct size

For histological analysis, we collected and embedded the MI (28 days post-MI) hearts in paraffin. Three sections of each heart (5 μm for each slide, two adjacent slides interval of 200 μm) were collected, starting from the apex to the base. The heart slices were then used for Masson staining to visualize the scar area. For scar size measurement, we measured the entire left ventricle area and the scar area for each slice with Image J, and the mean scar size of 3 slices was used as the scar size (%) for each heart.

### Neonatal primary cardiomyocytes isolation, culture and transfection

Neonatal ICR mice hearts were harvested at 1 day old (50–80 hearts each time). Neonatal hearts were chopped up, then transferred to a sterile flask for digestion with 20 ml of the digestive solution containing 0.06 g/100 ml trypsin (Sigma, United States) and 0.04 g/100 ml collagenase-II (Worthington, OH, United States) for 6–7 min each time. Cycle digestion until the myocardial tissue is completely digested into a cellular state. The cell suspension was resuspended using DMEM containing 10% FBS and incubated in an incubator for 45 min to remove cardiac fibroblasts. The remaining suspension was centrifuged with Percoll liquid (3,000 rpm, 30 min, slowly rising and falling), and the CMs of the middle layer were collected and cultured in an incubator with 5% CO_2_ at 37°C. After 24 h of culture, non-adherent cells were washed, and the remaining cells were cultured for another 24 h, then transfected with adenovirus for different experiments. To investigate the role of CDK9 in CMs and its effect on SGK3, we used the corresponding vector to transfect CMs for 48 h.

### Recombinant adenovirus

Recombinant adenovirus of CDK9, SGK3 (Ad5: cTNT-CDK9, SGK3) and adenovirus of control (Ad5: cTNT-CON) were designed by CMs specific cTNT promoter obtained from GeneChem Company (Shanghai, China). Adenoviruses carrying scrambled shRNA for mice CDK9, SGK3 (Ad5: cTNT-CDK9i, SGK3i) and adenovirus of control (Ad5: cTNT-CONi) were also purchased from GeneChem Company (Shanghai, China). The adeno-associated virus type 9 (AAV9) is driven by CMs specific cTNT promoter cTNT: 3Flag-CDK9 (AAV9: cTNT-CDK9) and control adeno-associated virus type 9 (AAV9: cTNT-CON) were also purchased from GeneChem Company (Shanghai, China). In neonatal primary CMs, the MOI (multiplicity of infection) of the effective transfection dose for viral transfection was 50 (9, 15).

### RNA extraction and quantitative real-time polymerase chain reaction

Total RNA was extracted using TRIzol reagent (Thermo Fisher Scientific) according to the manufacturer’s instructions. The quantity and quality of RNA were determined by Nanodrop 2000 spectrophotometer (Thermo Fisher Scientific). Then the PrimeScript RT Master Mix kit (Takara Bio, Kusatsu, Japan) was used to synthesize complementary DNA. Gene amplification was performed in Roche LightCycler 96 using specific primers and SYBR Green (Vazyme Biotech, Nanjing, China; Q131-02). The relative expression of the target genes was normalized to the expression level of 18S. The sequences of qRT-PCR primers are as follows: 18S-F: TAACGAACGAGACTCTGGCAT, 18S-R: CGGACATCTAAGGGCATCACAG; CDK9-F: TGAAGGCTGCGAATGTG, CDK9-R: GTTGGTGTATCGGT TGGG.

### Flow cytometry

At the required stage *in vitro*, primary neonatal mouse cardiomyocytes were washed and cultured with PBS, then 0.25% EDTA-free trypsin was added and cultured in a 37°C incubator for 5 min to digest the cells. The digestion was terminated with the complete medium containing 10% FBS; the cells were centrifuged (1,200 rpm, 5 min), washed with PBS and centrifuged again (1,200 rpm, 5 min). Afterward, the cell masses were resuspended in 70% ethanol and 20 μM filter dripping and fixing overnight at 4°C. The cells were centrifuged (1,200 rpm, 5 min), washed with PBS, and then centrifuged again (1,200 rpm, 5 min). The cells were resuspended in PI/RNase staining buffer (BD Biosciences, Franklin Lakes, NJ, United States) and incubated in a 37°C water bath for 40 min for flow cytometry detection. The data is generated by BD FACSVerse (Franklin Lakes, NJ, United States).

### Immunofluorescence staining

Hearts were fixed with 4% paraformaldehyde overnight and then embedded in paraffin. The paraffin-embedded hearts were cut into 5 μm slices and boiled in antigen retrieval buffer for 10 min. Then, the slices were immersed in PBS containing 0.2% Triton X-100 for 15 min and blocked with 5% bovine serum albumin for 2 h. Click-iT EdU imaging Kits (Thermo Fisher), anti-Ki67 antibody (1:200, Abcam; Ab16667) and anti-phosphorylation-histone 3 (pH3) antibody (1:200, CST; 9701) were used to culture with slices to identify cell cycle activity. Anti-troponin T (cTNT, 1:200, Abcam; Ab8295) was used to label CMs and Hoechst to label nuclei. Wheat germ agglutinin (WGA) staining (Thermo Fisher; w32466) was performed to stain the cell membrane. *In vitro*, neonatal mice CMs grown on 24-well plates were fixed with 4% paraformaldehyde for 30 min. After being blocked with 10% goat serum for 1 h, 5-ethynyl-2′-deoxyuridine (EdU), Ki67, and pH3 staining were performed. cTnT or a-Actinin were used to label CMs, and 4′, 6-Diamidino-2-phenylindole (DAPI) to label nuclei. Quantitative data were obtained by confocal microscopy (Zeiss, Oberkochen, Germany) and Carl Zeiss Microscopy (Jena, Germany). To detect CM proliferation, we calculated the number of EdU^+^, Ki67^+^, and pH3^+^ cardiomyocytes in the whole infarct border zone and their proportions in all border CMs.

### Western blot

For Western blot analysis, heart tissues or cells were lysed with lysis buffer (including 0.5% PMSF, 0.1% protease inhibitor, and 1% phosphatase inhibitor) (GeneChem, Shanghai, China). Cytoplasmic extracts and nuclear extracts were prepared using a nuclear and cytoplasmic extraction reagent kit (Thermo Fisher, United States). After determining the protein concentration by BCA Protein Assay, equal amounts of proteins were separated in SDS-PAGE gels and electro-transferred onto PVDF membranes (Millipore). And the membranes were incubated overnight at 4°C with primary antibodies (here, add specific primary antibody information) after blocking with bovine serum albumin (BSA). After washing three times for 30 min with TBST, membranes were cultured with the secondary antibodies diluted in blocking buffer for 2 h at room temperature. Quantification of band intensity was performed using Image J software (National Institutes of Health, Bethesda, MD, United States).

### Pull-down assay and mass spectrometry

The CMs treated with Ad5: cTNT-SGK3 were collected and lysed using RIPA lysis buffer (Beyotime, China). After detecting the concentration, the protein solution was divided into two groups and incubated overnight with IgG or SGK3 antibody at 4°C. The protein-antibody complex was captured using agarose beads (Millipore Corp., United States) for 4 h of rotation. SDS-PAGE was then used to separate the target proteins, and silver staining was used to identify the different bands between the two groups. The eluted protein solutions were analyzed through mass spectrometry analysis (Wuhan Genecreate Biological Engineering Co., Ltd.) using LC-MS/MS (ekspertTMnanoLC; AB Sciex TripleTOF 5600-plus) instrument.

### Co-immunoprecipitation

For the Co-IP assay, the Ad5: cTNT-SGK3 transfected CMs, neonatal and adult myocardial tissue were collected and lysed using RIPA lysis buffer (Beyotime, China). The proteins were incubated with SGK3 or CDK9 antibody overnight at 4°C. The protein-antibody complex was captured using PureProteome™ Protein A Magnetic Beads (Millipore Corp., United States) for 4 h of rotation. Then wash twice with RIPA lysis buffer and centrifugate to obtain the residue. Finally, the complex with loading buffer was boiled for 10 min and analyzed by Western blot.

### Statistical analysis

All data were statistically analyzed using SPSS 22.0 (IBM, Armonk, NY, United States) and expressed as mean ± SEM. The unpaired Student *t*-test was used to assess the statistical difference between the two groups. Comparisons between multiple groups were performed using either one-way ANOVA (using the Tukey multiple comparison test) or two-way ANOVA (using the *post hoc* Sidak test) to determine statistical differences. The results with *P*-values < 0.05 were considered statistically significant.

## Results

### CDK9 interacts with SGK3 and enhances SGK3 activity

**FIGURE 1:**
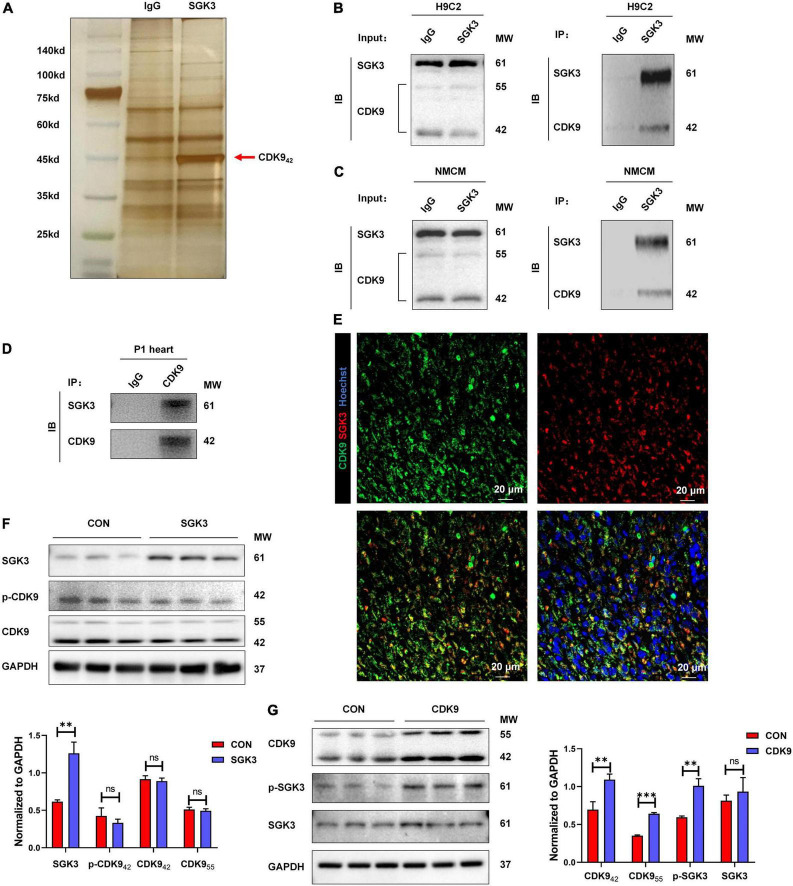
CDK9 interacts with SGK3 and enhances SGK3 activity. **(A–D)** Silver staining of a gel showing SGK3 and the control IgG pulldown proteins (CDK9, 42 kDa, red arrow) **(A)**. Co-immunoprecipitation reveals that CDK9_42_ interacts with SGK3 in the H9C2 cell line **(B)**, neonatal CMs **(C),** and P1 heart **(D)**. **(E)** Co-localization of SGK3 and CDK9 in neonatal myocardium was detected by immunofluorescence. **(F)** SGK3, phosphorylated (p-) CDK9 and CDK9 proteins were detected by Western blot in CMs transfected with Ad5: cTnT-SGK3 (MOI = 50). **(G)** CDK9, SGK3 Thr320 and SGK3 proteins were detected by Western blot in CMs transfected with Ad5: cTnT-CDK9 (MOI = 50). CMs indicate cardiomyocytes, MW indicates molecular weight, MOI indicates a multiplicity of infection; statistical significance was calculated with the unpaired Student *t*-test; data are presented as mean ± SEM, **P ≤ 0.01, ***P ≤ 0.001.

Our previous study found and verified the critical role of SGK3 in promoting endogenous cardiomyocyte proliferation via the GSK-3β/β-catenin pathway. To unravel the upstream signals of SGK3, we first selected 26 proteins detected to directly bind to SGK3 by pulldown and mass spectrometry analysis of SGK3 (Supplementary Figure 1 and Supplementary Tables 1, 2). Combined with previous studies, we screened out CDK9 as a potential SGK3-interacted kinase (Figure 1A). To verify the relationship between SGK3 and CDK9, we used IgG and SGK3 as baits to perform co-IP experiments in H9C2 cell lines and neonatal mice CMs, respectively. We detected significant CDK9 expression in the SGK3 group (Figures 1B,C). Given the high expression of CDK9 in neonatal hearts, we performed supplementary co-IP in P1 mice myocardium and obtained the same results (Figure 1D). Furthermore, the co-immunofluorescence images also indicated that CDK9 and SGK3 co-localized in the cytoplasm in the P1 myocardium (Figure 1E). Next, to clarify the exact relationship between SGK3 and CDK9, we overexpressed SGK3 or CDK9 in neonatal mice CMs, respectively. The results showed that overexpression of CDK9 could increase the phosphorylation activity of SGK3, while the intervention of SGK3 had no significant effect on the expression level and activity of CDK9 (Figures 1F,G).

### Expression pattern of CDK9 correlates with myocardial regeneration

**FIGURE 2:**
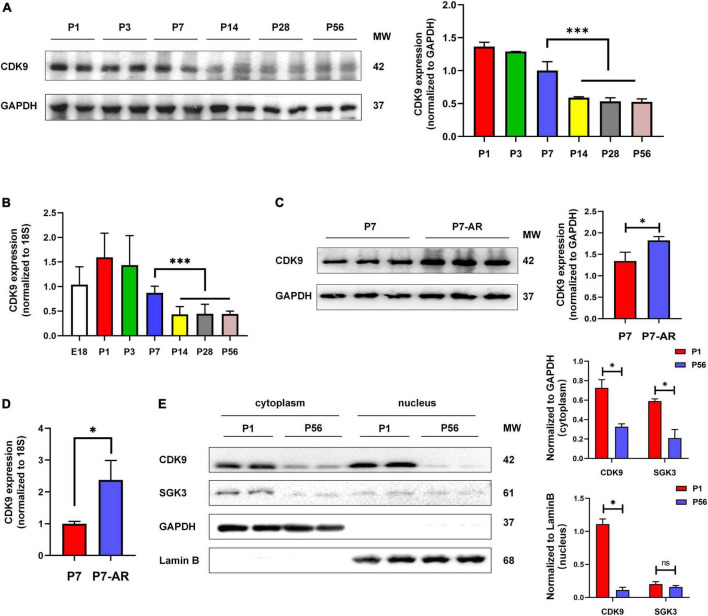
Expression pattern and subcellular localization of CDK9. **(A)** CDK9 proteins of ventricular myocardium detected by western blot in P1–P56 mice. **(B)** mRNA expression levels of CDK9 in embryo and P1–P56 mice. **(C)** CDK9 proteins of ventricular myocardium detected by western blot in P7 and P7-AR mice. **(D)** mRNA expression levels of CDK9 in P7 and P7-AR mice. **(E)** SGK3 and CDK9 proteins of ventricular myocardium in P1 and P56 mice submitted to nucleocytoplasmic fractionation. MW indicates molecular weight, AR indicates apical resection; statistical significance was calculated with the unpaired Student *t*-test; data are presented as mean ± SEM, *P ≤ 0.05, ***P ≤ 0.001; each dot indicates a biological replicate.

To verify whether CDK9 plays a vital role in myocardial regeneration in mice, we first examined the expression levels of CDK9 in myocardial tissues during the embryonic and postnatal period. The results showed that CDK9 was highly expressed during embryonic and 3 days after birth. While the expression level rapidly decreased after 7 days of life and was almost undetectable in the adult stage. This expression pattern matched the myocardial regeneration period (2, 3) (Figures 2A,B). In addition, we constructed AR in neonatal mice at P1 and found that CDK9 expression was significantly higher by western blot at P7 (Figures 2C,D). Next, to further reveal the expression characteristics of CDK9 from the dimension of time and space, we found by nucleocytoplasmic separation and western blot that CDK9 was markedly distributed in both the nucleus and cytoplasm, and the expression level was significantly higher at P1 day than P56 day. While the SGK3, as we previously revealed, was mainly distributed in the cytoplasm (Figure 2E).

### CDK9 regulates cardiomyocytes proliferation *in vitro*

**FIGURE 3:**
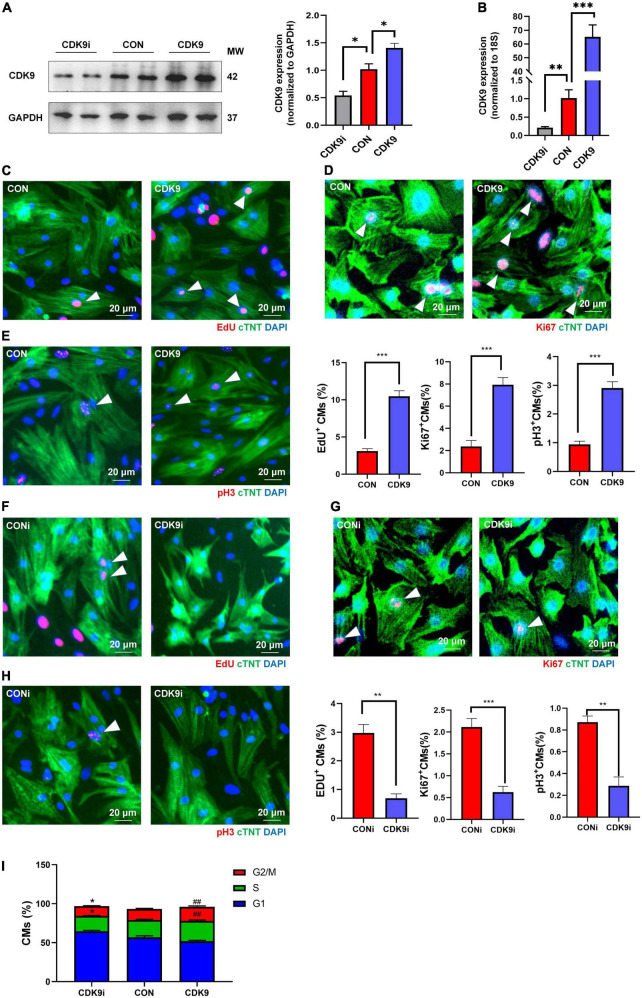
CDK9 promotes cardiomyocyte (CM) proliferation *in vitro*. **(A)** Knockdown and overexpression of CDK9 proteins confirmed by western blot in neonatal CMs treated with Ad5: cTnT-CON, Ad5: cTnT-CDK9 or Ad5: cTnT-CDK9 RNAi. **(B)** Knockdown and overexpression of CDK9 mRNA confirmed by qRT-PCR. **(C–E)** CM proliferation quantified in neonatal CMs transfected with Ad5: cTnT-CON or Ad5: cTnT-CDK9 by immunofluorescence for DNA synthesis **(C)**, cell-cycle activity **(D)**, and mitosis **(E)** (*n* = 6). **(F–H)** CM proliferation was quantified in neonatal CMs treated with Ad5: cTnT-CDK9 RNAi by immunofluorescence for DNA synthesis **(F)**, cell-cycle activity **(G)**, and mitosis **(H)** (*n* = 6). **(I)** CM cell cycle activity was detected by flow cytometry analysis after transfected with Ad5: cTnT-CON RNAi, Ad5: cTnT-CDK9, or Ad5: cTnT-CDK9 RNAi (CDK9i) (*n* = 3). White arrows in representative pictures point to the proliferating CMs; scale bars, 20 μm; CMs indicate cardiomyocytes, MW indicates molecular weight; statistical significance was calculated with the unpaired Student *t*-test; data are presented as mean ± SEM, *P ≤ 0.05, **P ≤ 0.01, ^##^*P* ≤ 0.01, ***P ≤ 0.001; each dot indicates a biological replicate.

Based on the importance of CMs proliferation in myocardial regeneration, we next isolated and purified primary CMs *in vitro* and verified the effect of CDK9 on CMs proliferation. We first demonstrated the transfection efficiency of Ad5: cTNT-CDK9i, Ad5: cTNT-CON and Ad5: cTNT-CDK9 in CMs via western blot and qRT-PCR (Figures 3A,B). Then we used immunofluorescence to detect cell proliferation indicators, such as EdU (DNA synthesis), Ki67 (cell cycle activity) and pH3 (mitosis). The results showed that overexpression of CDK9 could improve the proliferative capacity of CMs (Figures 3C–E), while knockdown of CDK9 impaired the proliferation ability of CMs (Figures 3F–H). In addition, flow cytometry analysis results also indicated that the proportion of G1-phase CMs decreased and S-phase and G2-phase CMs significantly increased after CDK9 overexpression, which was contrary in the CDK9 knockdown group (Figure 3I).

### CDK9 knockdown impairs myocardial regenerative repair after apical resection in neonatal mice

**FIGURE 4:**
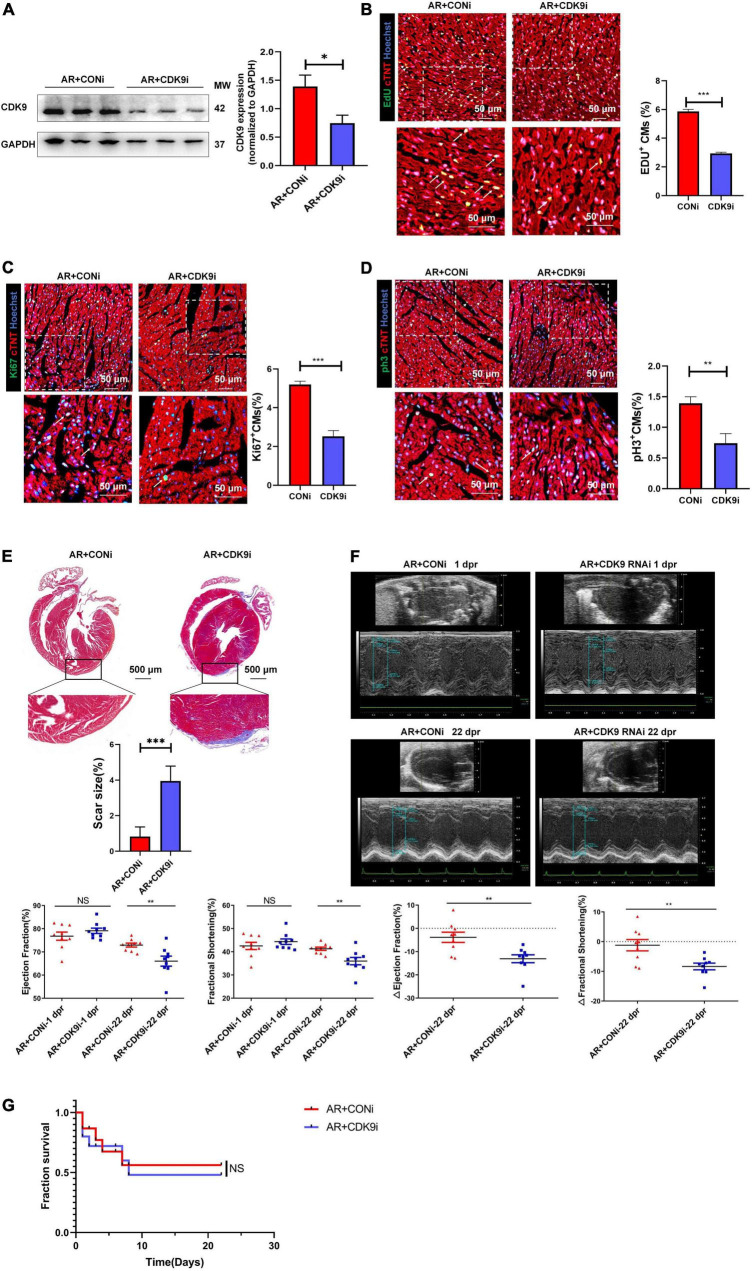
CDK9 knockdown impairs myocardial regeneration after AR in neonatal mice. **(A)** CDK9 protein of ventricular myocardium in neonatal mice injected Ad5: cTnT-CON RNAi or Ad5: cTnT-CDK9 RNAi (CDK9i) after AR surgery detected by western blot at 6 days after surgery. **(B–D)** CM proliferation in neonatal myocardial tissue after intra-myocardial injection with CDK9i or CONi quantified by immunofluorescence for DNA synthesis **(B)**, cell-cycle activity **(C)**, mitosis **(D)** (*n* = 3). **(E)** Comparison of scar size by Masson staining between the CONi and CDK9i groups at 22 days after AR surgery (*n* = 6). **(F)** LVEF and LVFS in P1 AR mice at 1 and 22 days after CDK9i or CONi injection was detected by echocardiography (*n* = 8). **(G)** Overall survival rate in neonatal mice from AR + CONi and AR + CDK9i groups (*n* = 15). Scale bars, 500 μm; CM indicates cardiomyocyte, MW indicates molecular weight; statistical significance was calculated with the unpaired Student *t*-test; data are presented as mean ± SEM, *P ≤ 0.05, **P ≤ 0.01, ***P ≤ 0.001; each dot indicates a biological replicate.

To validate the effect of CDK9 on myocardial regenerative repair in neonatal mice, we performed AR surgery on P1 mice. Normal neonatal mice can recover entirely after AR surgery because of their active CMs proliferation capacity (2). We took intra-myocardial injections of Ad5: cTNT-CDK9 RNAi or Ad5: cTNT-CONi after AR surgery in neonatal mice. Premixed EdU solution was intraperitoneally injected 4 days after AR (5 μg/mouse diluted in PBS). Western blot analysis at 6 days after surgery showed that the CDK9 expression level in the AR + CDK9i group was significantly lower than in the AR + CONi group (Figure 4A). Besides, cell proliferation staining (EdU, Ki67, and pH3) of apical region tissues from the two groups revealed that the proliferation efficiency of CMs in the AR + CDK9i group was significantly lower than that in the AR + CONi group (Figures 4B–D). Masson staining at 22 days after surgery showed that the degree of myocardial fibrosis in the AR + CONi group was significantly lower than that in the AR + CDK9i group (Figure 4E). To verify the effect of CDK9 knockdown on postoperative cardiac function in neonatal mice, we performed echocardiography in the two groups of mice at 1 and 22 days after AR surgery. The results showed that myocardial systolic function was impaired in both groups of mice at 1 day after surgery (LVEF: AR + CONi: 76.78 ± 1.772%, AR + CDK9i: 79.10 ± 1.143%, *p* = 0.724; LVFS: AR + CONi: 42.50 ± 1.549%, AR + CDK9i: 44.34 ± 1.186%, *p* = 0.755). In contrast, the recovery of cardiac function in the CDK9i group was significantly worse than in the CONi group at 22 days after surgery (LVEF: AR + CONi: 72.93 ± 0.851%, AR + CDK9i: 66.02 ± 2.160%, *p* = 0.0056; LVFS: AR + CONi: 41.28 ± 0.706%, AR + CDK9i: 35.97 ± 1.592%, *p* = 0.0034) (Figure 4F). The overall survival rate was similar between the AR + CONi and AR + CDK9i groups (Figure 4G).

### CDK9 overexpression promotes myocardial regenerative repair after myocardial infarction in adult mice

**FIGURE 5:**
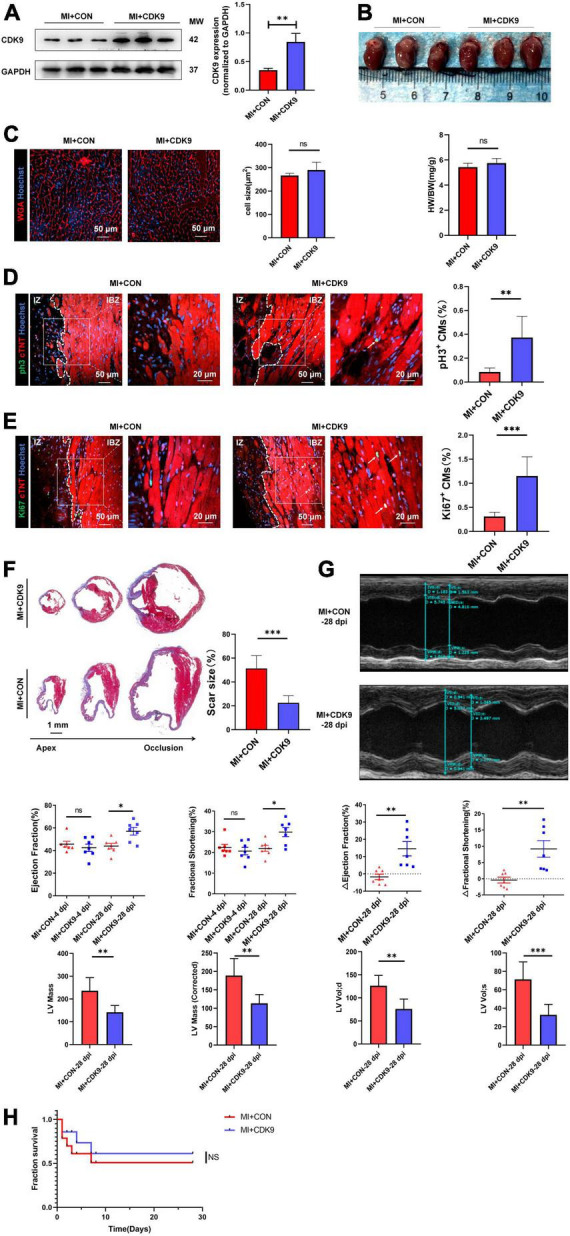
CDK9 overexpression promotes myocardial regeneration after MI in adult mice. **(A)** CDK9 protein of ventricular myocardium in adult mice injected with AAV9: cTnT-CDK9 or AAV9: cTnT-CON after MI detected by western blot (MI + CDK9 or MI + CON). **(B)** Left: cardiac morphology in adult mice subjected to MI + CDK9 or MI + CON at 14 dpi. Right: heart weight/Body weight (HW/BW) ratio in adult mice subjected to MI + CDK9 or MI + CON at 14 dpi (*n* = 6). **(C)** CM size in adult mice subjected to MI + CDK9 or MI + CON at 14 dpi was detected by wheat germ agglutinin (WGA) immunofluorescence (*n* = 6). **(D,E)** CM proliferation in adult myocardial tissue subjected to MI + CDK9 or MI + CON quantified by immunofluorescence for mitosis **(D)** and cell-cycle activity **(E)** (*n* = 6); white arrows point to the proliferating CMs. **(F)** Comparison of scar size by Masson staining between the CON and CDK9 groups at 28 days after MI (*n* = 6). **(G)** LVEF, LVFS, LV Mass and LV Vol d/s in adult MI mice at 4 and 28 days after CDK9 injection was detected by echocardiography (*n* = 7). **(H)** Overall survival rate in adult mice from MI + CON and MI + CDK9 groups (*n* = 14). Scale bars, 20/50 μm; CM indicates cardiomyocyte, MW indicates molecular weight, IZ indicates infarct zone, IBZ indicates infarcted border zone (<2 mm outside IZ); statistical significance was calculated with the unpaired Student *t*-test; data are presented as mean ± SEM, *P ≤ 0.05, **P ≤ 0.01, ***P ≤ 0.001; each dot indicates a biological replicate.

To explore the effect of CDK9 on injury repair and CMs proliferative capacity after MI in adult mice, we orthotopically injected AAV9: cTNT-CDK9 or AAV9: cTnT-CON at the same time after ligation of LAD in adult mice. We harvested samples 14 days after injection to verify successful CDK9 overexpression via western blot (Figure 5A). The heart size, heart weight/body weight ratio, and CM size were similar between the MI + AAV9: cTnT-CON and MI + AAV9: cTnT-CDK9 groups (Figures 5B,C). Then we confirmed the proliferation efficiency of CMs in the infarct border zone by immunofluorescence staining, which showed that the proliferative activity of CMs in the infarct border zone was significantly higher in the CDK9 overexpression group relative to the MI + AAV9: cTnT-CON group (Figures 5D,E). Meanwhile, Masson staining at 28 days after operation showed that the area of fibrosis in the infarct area was significantly ameliorative in the CDK9 overexpression group than in the CON group (Figure 5F). In addition, the results of echocardiography at 28 days after surgery showed that cardiac function (LVEF and LVFS) and structure (LV Mass and LV Vol d/s) was significantly improved in the CDK9 overexpression group (LVEF: 57.04 ± 8.80%; LVFS: 29.83 ± 5.75%) than in the CON group (LVEF: 43.94 ± 6.46%, *p* = 0.008; LVFS: 21.95 ± 3.75%, *p* = 0.010) (Figure 5G). The overall survival rate was similar between the MI + AAV9: cTnT-CON and MI + AAV9: cTnT-CDK9 groups (Figure 5H).

### CDK9 activates SGK3 and promotes cardiomyocytes proliferation via the GSK-3β/β-catenin pathway *in vitro*

**FIGURE 6:**
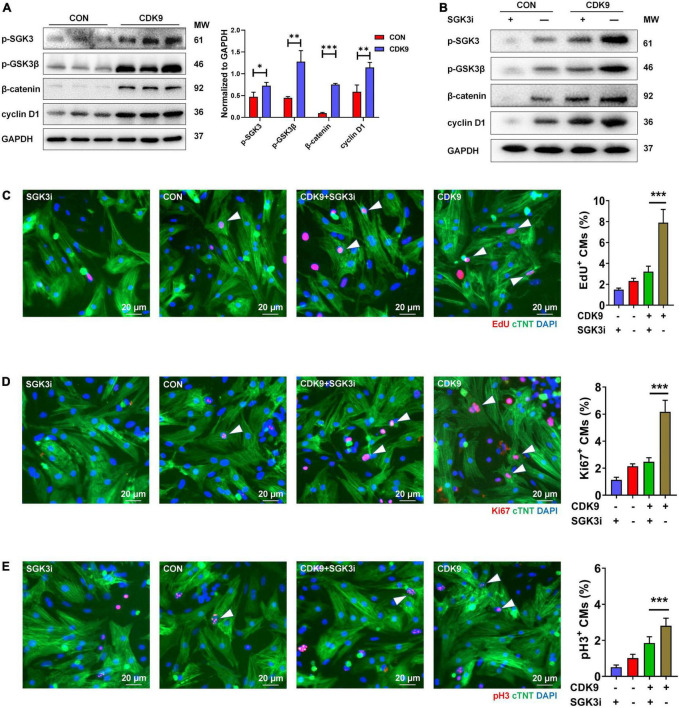
CDK9 regulates myocardial regeneration and repair through SGK3/GSK-3β/β-catenin axis *in vitro*. **(A)** p-SGK3, p-GSK3β, β-catenin and cyclin D1 proteins of ventricular myocardium in adult mice injected with Ad5: cTnT-CDK9 (CDK9) after MI detected by western blot. **(B)** p-SGK3, p-GSK3β, β-catenin and cyclin D1 proteins of neonatal CMs were detected by western blot after being transfected with CDK9 and/or Ad5:SGK3 RNAi (SGK3i). **(C–E)** CM proliferation is quantified by immunofluorescence for DNA synthesis **(C)**, cell-cycle activity **(D)**, and mitosis **(E)** in CMs transfected with CDK9 and/or SGK3i (*n* = 6). Scale bars, 20 μm; CMs indicate cardiomyocytes, MW indicates molecular weight; statistical significance was calculated with the unpaired Student *t*-test; data are presented as mean ± SEM, *P ≤ 0.05, **P ≤ 0.01, ***P ≤ 0.001; each dot indicates a biological replicate.

Based on our previous results, we have verified the binding of SGK3 and CDK9 and the effect of CDK9 on myocardial repair. Also, previous studies have shown that GSK-3β, β-catenin and cyclin D1 are downstream key targets of SGK3 (9). To further verify the effect of CDK9-SGK3 and its downstream signaling on proliferation in neonatal CMs, we examined relevant downstream including SGK3 after overexpression of CDK9 and found that overexpression of CDK9 significantly increased the phosphorylation levels of SGK3, significantly activated the phosphorylation site of GSK-3β at Ser9, and upregulated β-catenin and cyclin D1 expression (Figure 6A). Next, to verify whether CDK9 promotes CMs proliferation by regulating SGK3, we first examined the effect on CMs by intervening with CDK9 and SGK3 expression via western blot (Figure 6B). The immunofluorescence experiment showed that overexpression of CDK9 could promote CMs proliferation *in vitro*, and inhibition of SGK3 could weaken CMs proliferation ability. In contrast, simultaneous inhibition of SGK3 could partially attenuate the effect of CDK9 on CMs proliferation efficiency (EdU, Ki67, and pH3) (Figures 6C–E).

### CDK9 regulates myocardial repair through SGK3/GSK-3β/β-catenin axis *in vivo*

**FIGURE 7:**
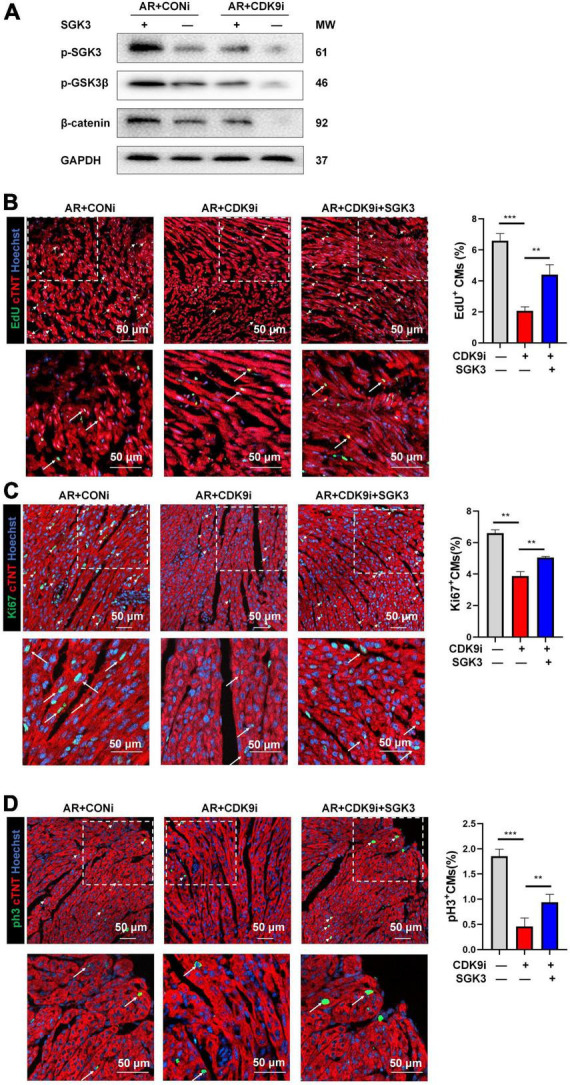
CDK9 regulates myocardial regeneration and repair through SGK3/GSK-3β/β-catenin axis *in vivo*. **(A)** p-SGK3, p-GSK3β and β-catenin proteins in neonatal myocardial tissue after intra-myocardial injection with Ad5: cTnT-CDK9 RNAi (CDK9i) and/or Ad5: cTnT-SGK3 (SGK3) after AR detected by western blot. **(B–D)** CM proliferation in neonatal AR myocardial tissue after intraperitoneal injection with CDK9i and/or SGK3 quantified by immunofluorescence for DNA synthesis **(B)**, cell-cycle activity **(C)**, mitosis **(D)** (*n* = 3). Scale bars, 50 μm; CMs indicates cardiomyocytes, AR indicates apical resection, MW indicates molecular weight; statistical significance was calculated with one-way ANOVA; data are presented as mean ± SEM, **P ≤ 0.01, ***P ≤ 0.001; each dot indicates a biological replicate.

After validating SGK3 as a critical target mediating CDK9 facilitated CMs proliferation *in vitro*, we needed to verify whether SGK3 mediated the function of CDK9 in promoting myocardial regenerative repair *in vivo*. We, therefore, constructed the mice AR model and orthotopically injected Ad5: cTNT-CDK9i simultaneously overexpressing SGK3. We first examined the effect on the myocardium by intervening with both CDK9 and SGK3 expression via western blot (Figure 7A). And the immunofluorescence results showed that the regenerative capacity of neonatal myocardium after AR was significantly impaired by CDK9 knockdown whereas partially alleviated by simultaneous SGK3 overexpression (Figures 7B–D).

### Research pattern diagram

**FIGURE 8:**
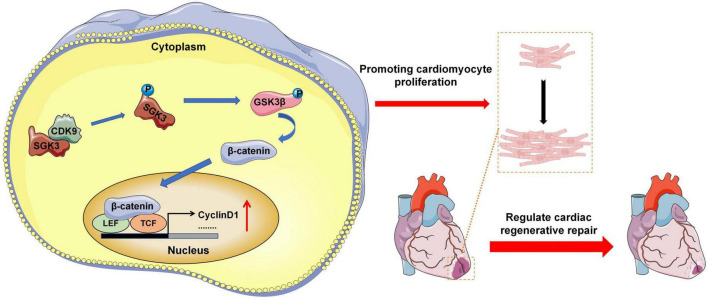
Research pattern diagram. Model depicting CDK9 binds to SGK3 and activates SGK3 and its downstream GSK-3β/β-catenin/cyclin D1 pathways to regulate the CM cycle activity, promoting CM proliferation and myocardial regeneration (CMs indicate cardiomyocytes).

Our experimental results indicate that CDK9 drives CMs cycle activity in mice myocardium and promotes CMs proliferation and myocardial repair by directly binding to and activating SGK3 and its downstream signaling (Figure 8).

## Discussion

The neonatal mammalian heart maintains regenerative capacity for a transient window, which declines rapidly after birth (16). The loss of the proliferative capacity of CMs involves alterations in gene expression and the activity of core regulatory components. SGK3 is a functional kinase we previously discovered and can promote CMs proliferation and cardiac repair after MI, while the underlying mechanism remains unknown (9). To uncover the upstream signals that regulate SGK3 and identify more potent key factors regulating myocardial regeneration, we performed a pulldown assay and mass spectrometry analysis of SGK3. We found CDK9 may have a pivotal role in CMs proliferation via interacting with SGK3. Therefore, CDK9 is expected to be a potential therapeutic target that can promote myocardial regeneration and repair by regulating SGK3.

It was reported that long-term overexposure to CDK9 may result in pathological cardiac hypertrophy; thus, CDK9 inhibitors have been investigated as new targets for treating hypertrophic cardiomyopathy (10). It is known that moderate hypertrophy of the myocardium after birth is regarded as a signature of myocardial maturation. Besides, recent studies have suggested that the proliferation and maturation of CMs are two sides of the same coin for myocardial regeneration, and the regulatory mechanisms of CMs proliferation and maturation have certain analogies: HIF1α can not only stimulate postnatal CMs to re-enter the cell cycle, but also promote CMs hypertrophy (17). Likewise, Igf1r is also thought to play an essential role in cardiac development and regenerative repair (18). Myocardial proliferation and physiological hypertrophy, as the crossroads of myocardial development and maturation, turn left or right, or even simultaneously, which is indeed an exciting truth to be further revealed (19). CDK9, which can regulate myocardial maturation, can also be used to explore the function of myocardial proliferation.

Gianfranco Matrone et al. showed that CDK9 plays a critical role in early cardiac development and CMs proliferation in zebrafish, and inhibition of CDK9 dephosphorylates its target site on the C-terminal domain of RNA polymerase II, thereby preventing regenerative repair after myocardial injury (20). Inhibition of CDK9 impairs the accumulation of neutrophils after injury and inhibits the recruitment of macrophages, thus hindering the repair of zebrafish heart damage. In contrast, transient inhibition of CDK9 can show positive effects (2-h window period) (21). In this study, we found that the expression pattern of CDK9 was consistent with the gradual loss of myocardial regenerative capacity *in vivo*, so we speculated that CDK9 might be a key regulator of myocardial regeneration. Our results indicated that the inhibition of CDK9 blocked CMs proliferation and myocardial repair after injury in neonatal mice. Exogenous overexpression of CDK9 significantly promoted CMs proliferation and regenerative repair in adult mice after MI.

CDK9 exists in two isoforms, the identified initially and more abundant one of 42kDa and the less abundant one of 55 kDa, the latter having an additional 117 aa at its N-terminus (22). These two isoforms are transcribed from the same *CDK9* gene but are composed of two different promoters. They are located more than 500 bp apart on the *CDK9* gene, of which the 42 kDa promoter is significantly more potent than the 55 kDa one (23). The expression of these two isoforms is differentially regulated in a signal-dependent and cell-type-specific manner. This study examined the expression of the two isoforms of CDK9 in primary CMs and myocardium under various physiological and pathological conditions. We found that the 42 kDa isoform plays a significant role in CMs proliferation. The classical part of CDK9 performs biological functions in the nucleus; CDK9 and cyclin T form a P-TEFb complex, which facilitates the transition from abortive to productive elongation by phosphorylating the CTD (C-terminal domain) of the large subunit of RNA polymerase II (RNAP II) POLR2A, SUPT5H and RDBP (22). CDK9 also regulates cytokine-induced transcriptional networks by promoting promoter recognition of target transcription factors (e.g., TNF-induced RELA/p65 activation and IL-6-induced STAT3 signaling), and plays a vital role in the genetics of cell growth, differentiation, and viral pathogenesis to promote RNA synthesis (4). However, little is known about the function of CDK9 in the cytoplasm, and the different subcellular localization of CDK9 is bound to mediate different modes of procedure. In the study of Zhao et al., function-guided proximity mapping unveils non-enzymatic PTMs in their non-canonical locales. CDK9 is hydroxynonenylated only in the cytoplasm and performs cross-compartment signaling (24).

This study demonstrates that CDK9 and SGK3 bind directly in the cytoplasm, and SGK3 is structurally highly homologous to AKT and can also regulate GSK-3β/β-catenin signaling (25). Activation of GSK-3β/β-catenin signaling is generally considered to be a common downstream pathway driving CMs proliferation and inhibiting CMs apoptosis (26, 27). In addition, our previous studies found that SGK3 can promote the recovery of cardiac function by inhibiting apoptosis. Therefore, the myocardial protective effect of CDK9 could be partly due to the inhibition of apoptosis by SGK3 (9). Previous studies have shown that Hippo/Yap, insulin-like growth factor (IGF), peroxisome proliferator-activated receptor δ (PPAR δ), neuregulin, and ERBB2 pathways are all involved in mediating the enhancement of downstream β-catenin signaling to stimulate CMs proliferation (28–31). GSK-3β/β-catenin pathway, as a part and essential loop of the Wnt signaling pathway, any intervention of arbitrary targets on this pathway could have a cascade amplifying effect on the target genes of the Wnt signaling pathway (32). Therefore, the further exploration of the relevant upstream signal initiators, such as Wnt ligands and Frizzled receptors, could bring more insights into the function of CDK9 in crosstalk between cardiomyocytes and other types of cells.

Moreover, we found that CDK9, as the upstream of SGK3, plays a critical role in promoting myocardial proliferation and cardiac repair after MI by activating the SGK3/GSK-3β/β-catenin pathway and even stimulates more pronounced CMs proliferation than SGK3. Notably, although our current findings suggest a positive effect of CDK9 on myocardial regenerative repair, appropriate treatment should be short-term and myocardial-specific, given the oncogenic property of CDK9. Myocardial regeneration is a holistic and dynamically regulated process, CMs proliferation is key to regenerating the lost myocardium following injury, which seems to be able to shape the cardiac microenvironment through the autonomous drive to achieve complete repair after injury (33, 34). Therefore, cardiomyocyte proliferation may play a guiding or triggering role in the process of myocardial regenerative repair, which needs further work to verify. Additionally, CDK9 inhibitors are widely used in clinical tumor treatment. The undesirable cardiotoxicity caused by CDK9 treatment requires extra vigilance, as the death of patients receiving targeted therapy is not limited to tumor occurrence and metastasis but fatal cardiovascular complications such as severe heart failure and malignant arrhythmia (35). Therefore, strategies targeting CDK9 would be cardio-oncology new research directions.

## Conclusion

Our study indicated that the expression pattern of CDK9 conforms to the changing trend of the mammalian myocardial regeneration period, and can interact with SGK3 to mediate cardiac repair and recovery after injury in the neonatal and adult heart through the GSK-3β/β-catenin pathway. The present work extends our knowledge of the kinase regulation in myocardial regeneration and indicates that targeting CDK9 and downstream signals might provide novel therapeutic implications in ischemic heart disease.

## Data availability statement

The datasets presented in this study can be found in online repositories. The names of the repository/repositories and accession number(s) can be found below: iProX (https://www.iprox.cn/), Project ID: IPX0004689000.

## Ethics statement

This animal study was reviewed and approved by Institutional Animal Care and Use Committee (IACUC) of Nanjing Medical University.

## Author contributions

JS, TY, and TW performed the experiments and analyzed the data. JS, TY, LZ, and LG wrote the manuscript. TS and JC provided experimental advice and some experimental supplies. LW designed the experiments and provided financial support. All authors contributed to the editing of the review and approved the final version of the manuscript for submission.

## Supplementary material

Supplementary Figure 1 A Venn diagram describing proteins/peptides identified by pulldown-MS analysis from IgG and SGK3 group.

Supplementary Table 1 Details of proteins/peptides detected in IgG, SGK3, or both.

Supplementary Table 2 Descriptions of 26 SGK3-binded proteins and their biological functions.

## Abbreviations

SGK3: threonine-protein kinase 3
CDK9: cyclin-dependent kinase 9
Co-IP: co-immunoprecipitation
P1: postnatal day 1
AR: apical resection
MI: myocardial infarction
CVD: cardiovascular disease
CMs: cardiomyocytes
CDKs: cyclin-dependent kinases
CDK4/6: CDK4 and CDK6 enzymes
HVSMCs: human vascular smooth muscle cells
IACUC: Institutional Animal Care and Use Committee
ICR: Institute of Cancer Research
SPF: specific pathogen-free
LVEF: left ventricular ejection fraction
LVFS: left ventricular fraction shortening
EdU: 5-ethynyl-2 ′ -deoxyuridine
DAPI: 4 ′ 6-diamidino-2-phenylindole
CTD: C-terminal domain
RNAP II: RNA polymerase II
IGF: insulin-like growth factor
PPAR δ: peroxisome proliferator-activated receptor δ .
