# Supplementary figures and images for: CDK9 binds and activates SGK3 to promote cardiac repair after injury via the GSK-3β/β-catenin pathway
Source: Front Cardiovasc Med. 2022 Aug 23;9:970745. doi: 10.3389/fcvm.2022.970745 (PMC9445272; doi:10.3389/fcvm.2022.970745)

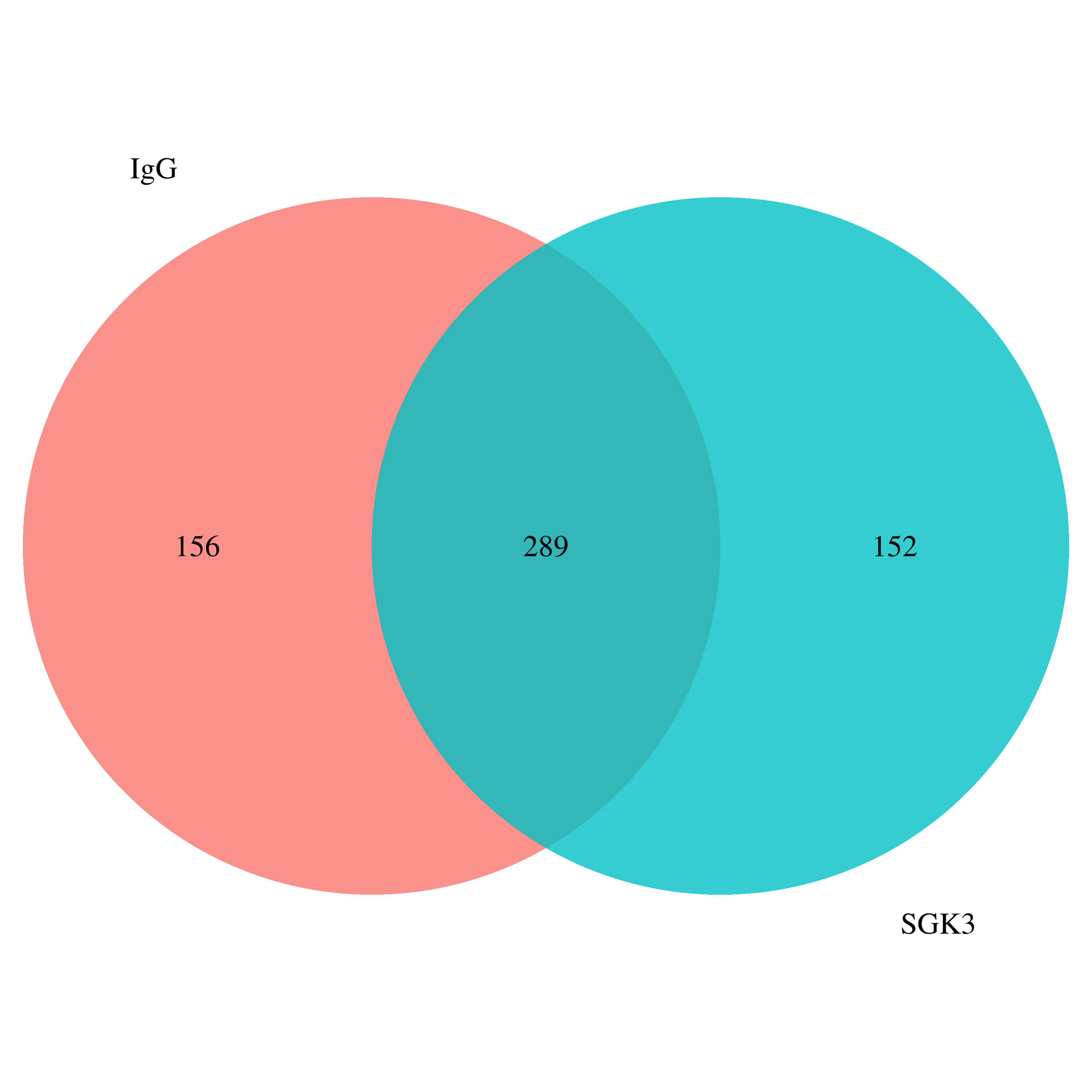

Supplement: Supplementary Figure 1 — A Venn diagram describing proteins/peptides identified by pulldown-MS analysis from IgG and SGK3 group. [file Image_1.PNG]
